# The effect of childhood trauma, ApoE genotype and HIV-1 viral protein R variants on change in cognitive performance

**DOI:** 10.1186/s13104-019-4869-9

**Published:** 2019-12-27

**Authors:** Jacqueline S. Womersley, Lara B. Clauss, Olivette Varathan, Susan Engelbrecht, Sian M. J. Hemmings, Soraya Seedat, Georgina Spies

**Affiliations:** 1 Department of Psychiatry, Faculty of Medicine and Health Sciences, Stellenbosch University, Francie van Zijl Drive, Tygerberg, 7505 South Africa; 2 South African Medical Research Council/Stellenbosch University Genomics of Brain Disorders Research Unit, Francie van Zijl Drive, Tygerberg, 7505 South Africa; 3 Joint Master in Neuroscience, Faculty of Life Sciences, University of Strasbourg, Strasbourg, France; 4 Division of Medical Virology, Faculty of Medicine and Health Sciences, Stellenbosch University, Francie van Zijl Drive, Tygerberg, 7505 South Africa; 5 National Health Laboratory Services (NHLS), Tygerberg Coastal, Tygerberg, Western Cape Region South Africa

**Keywords:** Apolipoprotein E, Childhood trauma, HIV-associated neurocognitive disorders, Viral protein R

## Abstract

**Objective:**

Gene–environment interactions contribute to the development of HIV-associated neurocognitive disorders. We examined whether childhood trauma, apolipoprotein E isoforms and viral protein R (Vpr) variants were associated with change in cognitive performance. Seventy-three seropositive women completed neuropsychological assessments at baseline and 1-year follow-up. We conducted genetic analyses using DNA obtained from blood and calculated risk scores based on Vpr amino acid 37, 41 and 55 variants that were previously associated with cognitive performance.

**Results:**

Global cognitive scores declined significantly over the 1-year study period (p = 0.029). A reduction in global cognitive scores was associated with childhood trauma experience (p = 0.039).

## Introduction

HIV-associated neurocognitive disorders (HAND) describe a spectrum of cognitive, behavioural and motor disturbances that may occur secondary to HIV infection [1]. Though improvement in and increased access to antiretroviral therapy (ART) has reduced the severity of HAND, milder forms persist and may affect up to 50% of the estimated 36.9 million people infected with HIV worldwide [1–3]. There is thus a need to identify genetic and environmental factors that increase the risk of developing HAND.

Genetic variation in the gene encoding apolipoprotein E (*ApoE*) has been suggested to contribute to HAND [4–6]. Two single nucleotide polymorphisms (rs7412 and rs429358) that encode C/T transitions give rise to cysteine/arginine amino acids at residue positions 112 and 158, the combination of which designates three isoforms i.e. ApoE ε2, ε3 and ε4 [7]. This genetic variation, specifically the ε4 isoform, may affect HIV infection [8] and replication in the brain [9], as well as susceptibility to HIV-related neurotoxic proteins [10]. Viral genetic variation may also contribute to HAND, with a study by Dampier et al. [11] identifying three amino acids (AAs) in viral protein R (Vpr) that affect neurocognitive performance. This 96-AA accessory protein plays a role in HIV infection and viral transcription, and may contribute to neuronal apoptosis, synaptic loss, proinflammatory signalling, oxidative stress and blood–brain barrier permeability [12–17]. Childhood trauma (CT) occurs during a period of rapid neurodevelopment and maturation, potentially exerting long-term consequences on neurobiology and mental health [18, 19]. Accordingly, CT has been identified as a predictor of poorer cognitive performance in women living with HIV [20–22].

The aetiology of HAND is complex and multifactorial. We examined the associative and interactive effects of CT and variation in *ApoE* and Vpr on cognitive function over a 1-year period in a cohort of South African women living with HIV.

## Main text

### Methods

#### Study design and participants

This was a nested study of an investigation of the biological endophenotypes of HIV and CT. Seventy-three women who met the study inclusion criteria [20] were recruited from community health care centres in Cape Town between 2008 and 2016. The study was approved by the Stellenbosch University Health Research Ethics Committee and written informed consent was obtained from all participants.

#### Clinical assessments

Participants provided sociodemographic data and were assessed for the presence of psychiatric disorders with the MINI-International Neuropsychiatric Interview-Plus [23]. Neurocognitive testing was done at baseline and 1-year with the HIV Neurobehavioral Research Center Neuropsychological (NP) battery [24], which taps into seven domains of cognitive function: motor ability, processing speed, verbal fluency, learning, delayed recall, attention/working memory and executive function. Raw scores from the seventeen NP tests were used to generate an age- and education-adjusted global cognitive z-score [20]. The 28-item Childhood Trauma Questionnaire-Short Form (CTQ-SF) was administered at baseline to assess for CT experienced prior to 18 years of age [25]. HIV infection was confirmed using enzyme-linked immunosorbent assays.

#### Apolipoprotein E genotyping

Genotyping for *ApoE* was performed as previously described [6]. DNA extracted from whole blood was subjected to KASP^®^ genotyping technology (LGC, Middelsex, UK) for the two *ApoE* variants rs7412 and rs429358. Genotyping was successfully undertaken for 62 participants.

#### Viral protein R amino acid assessment

Polymerase chain reaction (PCR) using DNA obtained from whole blood at 1-year follow-up and primers (Integrated DNA Technologies, Coralville, IO) flanking the region of interest was performed, and the resulting amplicons were cleaned using the Wizard SV Gel and PCR Clean-up System (Promega, Fitchburg, WI). Eluted samples were subjected to a sequencing PCR reaction using the BigDye Terminator v3.1 Cycle Sequencing kit (Applied Biosystems, Carlsbad, CA) and automated Sanger sequencing was performed on the 3130 × L Genetic Analyser (Applied Biosystems, Carlsbad, CA). Sequences were manually quality checked and assembled using Sequencher 5.4.6 (Gene Code Corporation, Ann Arbor, MI) with a minimum overlap of 1 and a minimum match of 60. Sequences were aligned and trimmed to contain Vpr bases using Geneious version 10.1.3 (Biomatters, Auckland, New Zealand). The trimmed sequences were submitted to Los Alamos National Laboratory for quality control and initial subtyping. Sequence subtyping was conducted using four online tools: Context-based Modelling for Expeditious Typing HIV-1 [26], jumping profile Hidden Markov Models [27], REGA HIV Subtyping Tool version 3 [28] and Recombinant Identification Program [29]. Vpr AA identification was successfully performed for 66 participants.

#### Statistical analyses

Demographic and clinical characteristics according to CT exposure (CTQ-SF score > 40 indicative of at least mild-to-moderate CT exposure) were assessed using Student’s t-tests, Mann–Whitney U tests or Pearson Chi square tests. As viral load data were highly skewed, we generated log-transformed data for subsequent analyses. Genotyping results were used to classify participants according to the presence of the ε4 isoform i.e. hetero- and homozygous individuals were grouped. We classified AAs according to their effects on cognition, as reported by Dampier et al. [11], with higher scores indicative of greater risk. Accordingly, the AAs were scored as follows: AA37 I (neuroprotective) variant = 0, other (neutral) variants = 1; AA41 S (neuroprotective) variant = 0, N (risk) variant = 2, other (neutral) variants = 1; and AA55 A (risk) variant = 1, other (neutral) variants = 0. A composite risk score was generated by summing the values assigned to the three AAs. Repeated measures analyses with 1-year global cognitive score as the outcome measure, baseline global cognitive score as a predictor and continuous CTQ-SF scores, ApoE ε4 carrier status, and individual and composite AA risk scores as between subject factors in regression models were performed. We examined whether including interactions between predictor variables explained more of the variation in our data by using ANOVA to compare the fit of the models produced using factors or factors plus their interaction effects. As CD4 and CD8 lymphocyte counts, as well as both original and log-transformed viral load values were not associated with global cognitive scores, these parameters were not included as covariates. All statistical analyses were performed in R [30] and an alpha value of less than 0.05 was deemed statistically significant.

### Results

#### Demographic and clinical characteristics

Neurocognitive assessments at baseline and follow-up were completed by 73 women (mean age 35.23 years) who self-identified as “Black, isiXhosa-speaking”. Approximately three-quarters of the sample (76.7%) were on ART. Based on the 63 women who provided their year of diagnosis, the mean time since confirmed HIV infection was 13.13 years. A repeated measures *t* test comparing global cognitive scores at baseline (mean = − 0.02, standard deviation = 0.55) and follow-up (mean = − 0.12, standard deviation = 0.53) indicated a significant decline in cognitive scores over the 1-year study period (t(72) = 2.22, p = 0.029). Most participants (n = 54, 74%) self-reported CT of at least low-to-moderate severity (CTQ-SF ≥ 41). Participants who experienced CT were more likely to be on ART (p = 0.001) and showed trends towards a lower median level of education (p = 0.052) and lower follow-up global cognitive score (p = 0.064). Our assessment of viral load was limited to the 38 individuals (15 without CT and 23 with CT) with a viral load above the detectable level i.e. ≥ 40 copies/ml. Only analyses that used the original data indicated significantly higher viral loads in participants with CT exposure (p = 0.023). Demographic and clinical data are summarised in Table 1. The CTQ-SF scores ranged from 25 to 114 and were used to categorise participants based on the severity of CT exposure (Table 2).

**Table 1:** Baseline demographic and clinical characteristics of study participants by childhood trauma exposure

	All participants (n = 73)	Childhood trauma (n = 54)	No childhood trauma (n = 19)	t	U	χ^2^	P
Mean age in years ± SD	35.23 ± 7.24	35.81 ± 6.60	33.58 ± 8.80)	–1.16			0.250
Median years of education (interquartile range)	11.00 (9.00 –11.00)	10.00 (9.00 –11.00)	11.00 (10.00–12.00)		362.00		0.052
Median viral load (interquartile range)^a^	18,459.50 (1732.25–64,831.75)	24,510.00 (4478.00–64,634.50)	9759.00 (1284.50–50,905.50)		320.50		0.023
Median log-transformed viral load (interquartile range)^a^	4.26 (3.20 –4.82)	4.39 (3.52 –4.81)	3.99 (3.08–4.87)		140.00		0.332
Median CD4 count (interquartile range)	453.00 (270.50–664.25)	432.00 (259.50–643.75)	481.50 (410.25–677.75)		412.00		0.336
Median CD8 count (range)	834.00 (620.50–1107.75)	768.00 (539.00–1090.00)	1050.00 (754.00–1284.00)		357.50		0.203
On ART treatment	56	47	9			12.38	0.001
AA37 ^b^						1.11	0.574
I	3	3	0				
AA41^b^						2.78	0.427
S	24	20	4				
N	12	7	5				
AA55^b^						5.61	0.061
A	13	13	0				
Hetero- or homozygous for the ApoE ε4 allele^c^	26	18	8			1.72	0.190
Mean baseline global cognitive score ± SD	–0.02 ± 0.55	–0.08 ± 0.54	0.15 ± 0.53	1.61			0.112
Mean follow-up global cognitive score ± SD	–0.12 ± 0.53	–0.19 ± 0.55	0.07 ± 0.41	1.88			0.064

*AA* amino acid, *ApoE* apoliprotein E, *ART* antiretroviral therapy, *SD* standard deviation

Parametric and non-parametric data are represented as mean ± SD and median and interquartile range respectively

^a^Viral load comparisons included the 38 participants with a viral load of greater than or equal to 40, the lower limit of sensitivity of the assay

^b^Amino acid data was available for 66 participants

^c^ApoE ε4 allele comparison included the 63 participants for whom genotype information was available

**Table 2:** Participants categorisation by childhood trauma severity

	None or minimal CTQ-SF ≤ 40	Low-to-moderate 41 ≤ CTQ-SF ≤ 55	Moderate-to-severe 56 ≤ CTQ-SF ≤ 72	Severe-to-extreme 73 ≤ CTQ-SF
Number of participants (%)	19 (26.0)	10 (13.7)	17 (23.3)	27 (37.0)

*CTQ-SF* Childhood Trauma Questionnaire-Short Form

#### Change in global cognitive score analyses

**Fig. 1:**
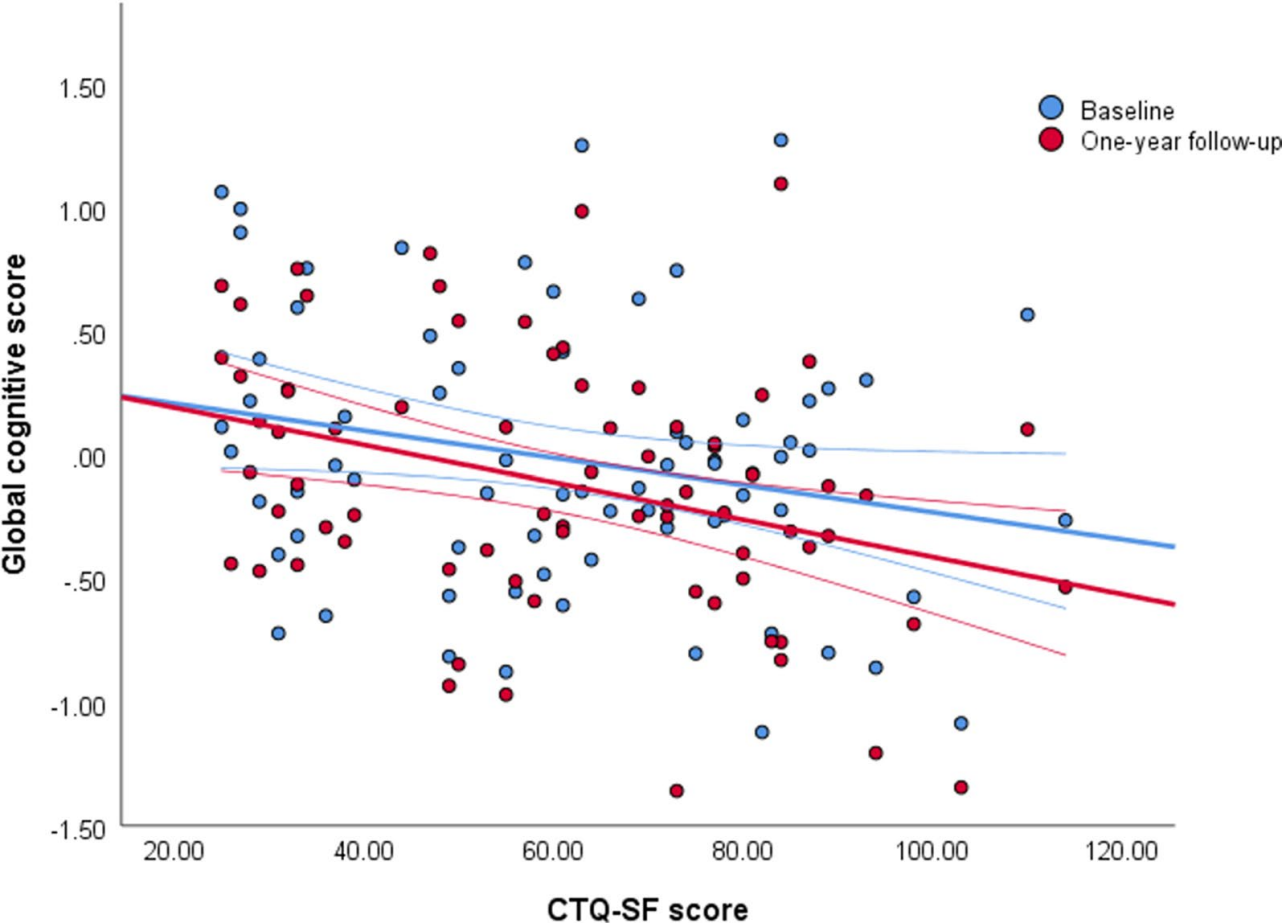
Childhood trauma was significantly associated with decreased global cognitive scores over the 1-year study period. Each unit increase in CTQ-SF score was associated with a 3.88  ×  10^− 3^ decrease in global cognitive score (p = 0.039)

Repeated measures analyses indicated that CTQ-SF score was significantly associated with decreased global cognitive scores over the 1-year study period with each unit increase in CTQ-SF score associated with a 3.88 × 10^− 3^ decrease in global cognitive score (p = 0.039) (Fig. 1). No significant effects of ApoE isoform or AA risk variants on global cognitive function were found. Test statistics for study variables are summarised in Additional file 1: Table S1. Comparing model fit revealed that including the interactive effects of *ApoE4*, CT, AA41 and AA55 explained significantly more of the variance in cognitive function (Akaike information criterion reduced from 56.28 to 54.69, p = 0.042). Factor interaction effects on model fit are summarised in Additional file 2: Table S2.

## Discussion

Our study examined the contribution of variation in *ApoE* and Vpr to change in cognitive function over time in a unique cohort of South African women with variable exposure to CT. In keeping with previous studies, we found that CT experience was associated with a decline in global cognitive scores over the course of one year [20, 21]. Though the estimated effect of CTQ-SF score on global cognitive score was small, it is possible that the influence of high CT exposure acting over a prolonged period may produce clinically meaningful effects. Drawing on the findings of Dampier et al. [11], we generated risk scores based on Vpr AA variation. Our analyses indicated that including the interactive effects of *ApoE*, CTQ score and AA41 and AA55 risk variants explained significantly more of the variance in global cognitive scores over the 1-year study period. Such higher order interactive effects may be due to common pathophysiological mechanisms shared by these factors, such as inflammation, oxidative stress, and altered synaptic plasticity and glucocorticoid signalling, which are implicated in HAND pathogenesis [15, 16, 31–39]. These factors may interact to facilitate disease progression. For example, Vpr influences both host and viral gene transcription through its role as a coactivator of glucocorticoid receptors [40]. Thus, CT-induced changes to glucocorticoid receptor mRNA expression may in turn affect HIV pathophysiology through a Vpr-dependent mechanism [41]. The ApoE ε4 isoform has been suggested to mediate the effects of CT on later life mental health and cognition [42, 43]. Finally, both AA41 and AA55 are included in the second alpha helix of Vpr, a region of the protein associated with activation of viral transcription as well as cellular apoptosis, both of which are likely important in HIV-related neuropathogenesis [44]. Though our results are in keeping with the multifactorial aetiology of HAND, further studies in larger sample sizes will be required to unpack these interactions, determine their effect size and ultimately their clinical significance.

## Limitations

The NP battery provides information on seven cognitive domains and thus our use of a global score will not reveal domain-specific effects. Though we found a reduction in cognitive scores over one year, the clinical significance of this decline is uncertain as both baseline and follow-up cognitive scores were within normal range. Nevertheless, the drop in scores is concerning considering that most women were on ART. Several confounding factors potentially affect CT measurement. As a retrospective self-report measure, the CTQ-SF may be influenced by subjective factors such as memory and recall bias [45]. We used the CTQ-SF total score in our regression models and therefore cannot determine whether specific subtypes of CT produce differential effects on cognitive performance. It is possible that additional covariates not included in our study, such as HIV-related comorbidities, may affect cognitive scores. Our findings are also not necessarily applicable to men. We grouped individuals hetero- and homozygous for the ε4 isoform, and thus cannot determine whether dose–response effects could influence global cognitive scores. Finally, our relatively small sample size may be insufficient to identify relationships between our predictor variables and change in cognitive function, especially given the rarity of certain genetic variants, such as AA37 I, in our study group.

Nevertheless, these findings support the influence of CT on neurocognitive function and suggest that host and viral genetic factors interact to influence cognitive function in HIV. This study has several strengths. We used longitudinal data, an important consideration in examining the development of disorders. Our study was conducted in a population that is disproportionately affected by HIV. Females comprise the majority of the approximately 7.52 million seropositive individuals in South Africa [46]. Furthermore, CT is common in South Africa, including among people living with HIV [47, 48]. We suggest future studies explore the contributions of *ApoE*, Vpr and CT to HAND, using larger sample sizes, which include both men and women.

## Supplementary information

**Additional file 1: Table S1.** Predictive value of variables on one-year global cognitive scores.

**Additional file 2: Table S2.** Predictive value of interactions on one-year global cognitive scores.

## Data Availability

The datasets generated and/or analysed during the current study are available from the corresponding author on reasonable request.

## Abbreviations

AA: amino acid
ApoE: apolipoprotein E
ART: antiretroviral therapy
CT: childhood trauma
CTQ-SF: Childhood Trauma Questionnaire-Short Form
HAND: HIV-associated neurocognitive disorders
NP: neuropsychological
PCR: polymerase chain reaction
Vpr: viral protein R
