# Supplementary material for: The effect of childhood trauma, ApoE genotype and HIV-1 viral protein R variants on change in cognitive performance
Source: BMC Res Notes. 2019 Dec 27;12:828. doi: 10.1186/s13104-019-4869-9 (PMC6935155; doi:10.1186/s13104-019-4869-9)
Supplement: Supplementary file 1 — Additional file 1: Table S1. Predictive value of variables on one-year global cognitive scores. [file 13104_2019_4869_MOESM1_ESM.docx]

Additional Table S1: Predictive value of variables on one-year global cognitive scores

| Model coefficient | Estimate | Std. error | t value | Pr (>\|t\|) |
| --- | --- | --- | --- | --- |
| CTQ-SF score | -3.88 x 10^-3^ | 1.84 x 10^-3^ | -2.10 | 0.039 |
| Viral load | -2.05 x 10^-7^ | 6.88 x 10^-7^ | -0.30 | 0.768 |
| Log-transformed viral load | 4.00 x 10^-3^ | 0.03 | 0.16 | 0.871 |
| CD8 count | -3.51 x 10^-5^ | 6.31 x 10^-5^ | -0.56 | 0.580 |
| CD4 count | 1.07 x 10^-4^ | 1.55 x 10^-4^ | 0.69 | 0.494 |
| ApoE4 isoform carrier | -9.28e x 10^-4^ | 0.09 | -0.01 | 0.992 |
| AA37 | 0.17 | 0.22 | 0.80 | 0.428 |
| AA41 | -0.07 | 0.07 | -1.13 | 0.263 |
| AA55 | -0.08 | 0.11 | -0.68 | 0.496 |
| AA composite risk score | -0.06 | 0.06 | -1.09 | 0.279 |

AA = amino acid; ApoE4 = apolipoprotein E; CTQ-SF = Childhood Trauma Questionnaire – Short Form. All models included baseline global cognitive score as a predictor variable.
