# Supplementary material for: The effect of childhood trauma, ApoE genotype and HIV-1 viral protein R variants on change in cognitive performance
Source: BMC Res Notes. 2019 Dec 27;12:828. doi: 10.1186/s13104-019-4869-9 (PMC6935155; doi:10.1186/s13104-019-4869-9)
Supplement: Supplementary file 2 — Additional file 2: Table S2. Predictive value of interactions on one-year global cognitive scores. [file 13104_2019_4869_MOESM2_ESM.docx]

Additional Table S2: Predictive value of interactions on one-year global cognitive scores

| Factors | Degrees of freedom of residuals | Deviance of Residuals | Degrees of freedom | Dev | Pr(>Chi) |
| --- | --- | --- | --- | --- | --- |
| ApoE4 x AA37 | | | | | |
| ApoE4 + AA37 | 53 | 7.45 |  |  |  |
| ApoE4 * AA37 | 52 | 7.38 | 1 | 0.07 | 0.470 |
| ApoE4 x AA41 | | | | | |
| ApoE4 + AA41 | 53 | 7.41 |  |  |  |
| ApoE4 * AA41 | 52 | 7.25 | 1 | 0.16 | 0.283 |
| ApoE4 x AA55 | | | | | |
| ApoE4 + AA55 | 53 | 7.47 |  |  |  |
| ApoE4 * AA55 | 52 | 7.32 | 1 | 0.15 | 0.300 |
| ApoE4 x AA composite risk score | | | | | |
| ApoE4 + AA composite risk score | 53 | 7.41 |  |  |  |
| ApoE4 * AA composite risk score | 42 | 7.22 | 1 | 0.19 | 0.239 |
| ApoE4 x CTQ-SF | | | | | |
| ApoE4 + CTQ-SF | 58 | 7.58 |  |  |  |
| ApoE4 * CTQ-SF | 57 | 7.57 | 1 | 3.00 x 10^-3^ | 0.874 |
| ApoE4 x AA37 x AA41 | | | | | |
| ApoE4 + AA37 + AA41 | 52 | 7.35 |  |  |  |
| ApoE4 * AA37 * AA41 | 49 | 7.09 | 3 | 0.26 | 0.612 |
| ApoE4 x AA37 x AA55 | | | | | |
| ApoE4 + AA37 + AA55 | 52 | 7.41 |  |  |  |
| ApoE4 * AA37 * AA55 | 49 | 7.20 | 3 | 0.21 | 0.694 |
| ApoE4 x AA41 x AA55 | | | | | |
| ApoE4 + AA41 + AA55 | 52 | 7.35 |  |  |  |
| ApoE4 * AA41 * AA55 | 48 | 6.65 | 4 | 0.70 | 0.283 |
| ApoE4 x AA37 x AA41 x CTQ-SF | | | | | |
| ApoE4 + AA37 + AA41 + CTQ-SF | 51 | 7.01 |  |  |  |
| ApoE4 * AA37 * AA41 * CTQ-SF | 45 | 6.47 | 6 | 0.54 | 0.713 |
| ApoE4 x AA37 x AA55 x CTQ-SF | | | | | |
| ApoE4 + AA37 + AA55 + CTQ-SF | 51 | 7.13 |  |  |  |
| ApoE4 * AA37 * AA55 * CTQ-SF | 45 | 6.83 | 6 | 0.31 | 0.918 |
| ApoE4 x AA41 x AA55 x CTQ-SF | | | | | |
| ApoE4 + AA41 + AA55 + CTQ-SF | 51 | 7.01 |  |  |  |
| ApoE4 * AA41 * AA55 * CTQ-SF | 41 | 4.80 | 10 | 2.21 | 0.042 * |
| CTQ-SF x AA37 | | | | | |
| CTQ-SF + AA37 | 62 | 7.98 |  |  |  |
| CTQ-SF * AA37 | 61 | 7.93 | 1 | 0.05 | 0.543 |
| CTQ-SF x AA41 | | | | | |
| CTQ-SF + AA41 | 62 | 7.76 |  |  |  |
| CTQ-SF * AA41 | 61 | 7.76 | 1 | 2.48 x 10^-3^ | 0.889 |
| CTQ-SF x AA55 | | | | | |
| CTQ-SF + AA55 | 62 | 7.99 |  |  |  |
| CTQ-SF * AA55 | 61 | 7.98 | 1 | 0.01 | 0.756 |
| CTQ-SF x AA composite risk score | | | | | |
| CTQ-SF + AA composite risk score | 62 | 7.83 |  |  |  |
| CTQ-SF * AA composite risk score | 61 | 7.81 | 1 | 0.02 | 0.730 |
| CTQ-SF x AA37 x AA41 | | | | | |
| CTQ-SF + AA37 + AA41 | 61 | 7.75 |  |  |  |
| CTQ-SF * AA37 * AA41 | 58 | 7.69 | 3 | 0.08 | 0.892 |
| CTQ-SF x AA37 x AA55 | | | | | |
| CTQ-SF + AA37 + AA55 | 61 | 7.97 |  |  |  |
| CTQ-SF * AA37 * AA55 | 58 | 7.90 | 3 | 0.07 | 0.910 |
| CTQ-SF x AA41 x AA55 | | | | | |
| CTQ-SF + AA41 + AA55 | 61 | 7.76 |  |  |  |
| CTQ-SF * AA41 * AA55 | 57 | 7.53 | 4 | 0.23 | 0.784 |
| CTQ-SF x ApoE4 x AA composite risk score | | | | | |
| CTQ-SF + ApoE4 + AA composite risk | 52 | 7.05 |  |  |  |
| CTQ-SF * ApoE4 * AA composite risk | 48 | 6.69 | 4 | 0.36 | 0.635 |

AA = amino acid; ApoE4 = apolipoprotein E; CTQ-SF = Childhood Trauma Questionnaire – Short Form. All models included baseline global cognitive score as a predictor variable.
